# Hereditary pulmonary arterial hypertension burden in pediatrics: A single referral center experience

**DOI:** 10.3389/fped.2023.1050706

**Published:** 2023-03-29

**Authors:** Maki Ishizuka, Wenxin Zou, Elise Whalen, Erin Ely, Ryan D. Coleman, Dolores H. Lopez-Terrada, Daniel J. Penny, Yuxin Fan, Nidhy P. Varghese

**Affiliations:** ^1^ Department of Pediatrics, Critical Care, Baylor College of Medicine, Texas Children’s Hospital, Houston, TX, United States; ^2^ Department of Pathology & Immunology, Baylor College of Medicine, Houston, TX, United States; ^3^ John Welsh Cardiovascular Diagnostic Laboratory, Division of Genomic Medicine, Department of Pathology, Texas Children’s Hospital, Houston, TX, United States; ^4^ Department of Pediatrics, Pulmonology, Baylor College of Medicine, Texas Children’s Hospital, Houston, TX, United States; ^5^ Division of Genomic Medicine, Department of Pathology, Texas Children’s Hospital, Houston, TX, United States; ^6^ Department of Pediatrics, Cardiology, Baylor College of Medicine, Texas Children’s Hospital, Houston, TX, United States; ^7^ Departments of Pathology & Immunology and Pediatrics-Cardiology, Baylor College of Medicine, Houston, TX, United States

**Keywords:** pediatrcis, pulmonary arterial hypertension, hereditary, tertiary referral center, genetic

## Abstract

**Introduction:**

Hereditary pulmonary arterial hypertension (HPAH) is a rare yet serious type of pulmonary arterial hypertension (PAH). The burden in the pediatric population remains high yet underreported. The objective of this study is to describe the distribution of mutations found on targeted PAH panel testing at a large pediatric referral center.

**Methods:**

Children with PAH panel administered by the John Welsh Cardiovascular Diagnostic Laboratory at Texas Children's Hospital and Baylor College of Medicine in Houston, Texas between October 2012 to August 2021 were included into this study. Medical records were retrospectively reviewed for clinical correlation.

**Results:**

Sixty-six children with PAH underwent PAH genetic testing. Among those, 9 (14%) children were found to have pathogenic mutations, 16 (24%) children with variant of unknown significance and 41 (62%) children with polymorphism (classified as likely benign and benign). BMPR2 mutation was the most common pathogenic mutation, seen in 6 of the 9 children with detected mutations. Hemodynamic studies showed higher pulmonary vascular resistance among those with pathogenic mutations than those without (17.4 vs. 4.6 Wood units). All children with pathogenic mutations had severe PAH requiring triple therapy. There were tendencies for higher lung transplantation rate but lower mortality among those with pathogenic mutations.

**Conclusions:**

Abnormalities on genetic testing are not uncommon among children with PAH, although majority are of unclear significance. However, children with pathogenic mutations tended to present with more severe PAH requiring aggressive medical and surgical therapies. Genetic testing should be routinely considered due to consequences for treatment and prognostic implications. Larger scale population studies and registries are warranted to characterize the burden of HPAH in the pediatric population specifically.

## Introduction

Heritable pulmonary arterial hypertension (HPAH) is a rare yet serious type of pulmonary arterial hypertension (PAH). Although adult epidemiologic studies report the median age of diagnosis for PAH is currently in the 3rd–4th decade of life (1, 2), age of diagnosis for HPAH is extremely variable. Emerging clinical and genetic data indicate that there are fundamental differences between pediatric and adult-onset disease (3, 4), reflecting the influence of epigenetic and possibly environmental factors. There is a greater genetic burden in children with genetic factors contributing to 42% of pediatric onset PAH compared to 13% of adult-onset PAH (3). At present, mutations in 16 genes have been linked to development of HPAH, many of them involved in or affecting the BMPR2 (Bone morphogenetic protein receptor 2) signaling pathway (3, 5). BMPR2 is highly expressed on pulmonary vascular endothelium and mutations in BMPR2 comprise the 70%–80% of HPAH cases (6). The co-factors of BMPR2 endoglin (ENG) and activin receptor like kinase 1 (ACVRL1) are predominantly altered in hereditary hemorrhagic telangiectasia-associated PAH (7, 8). Mutations in the BMP receptor type IA and type 1B [BMPR1A and BMPR1B, also called activin receptor-like kinase 6 (ALK6)], caveolin-1 (CAV1), eukaryotic initiation translation factor 2 alpha kinase 4 (EIF2AK4), potassium two-pore-domain channel subfamily K member 3 (KCNK3), SMAD family members 4 and 9 (SMAD4 and SMAD9), and T-box 4 (TBX4) have all been identified as less frequent or rare causes of PAH (9).

Pediatric PAH differs from adult-onset PAH in many important aspects, including clinical presentation, etiology, genetic burden, and specific genes involved. In pediatric-onset PAH, transcription factors TBX4 and SOX17 are seen with high frequency following BMPR2 (3). TBX4 and SOX 17 are not expressed in pulmonary arterial endothelial cells or smooth muscles, but in embryonic tissues and have prominent roles in lung and vasculature development (10, 11). De novo variants are a frequent characteristic of pediatric-onset PAH, contributing to 15% of patient population (3). These discoveries have prompted considerable development of targeted gene mutation panels for evaluation of pediatric PAH patients. Here we describe the distribution of mutations in a pediatric PAH population of a large referral center.

## Materials and methods

### Patient population

This was a single center study. Our institution is the largest children's hospital in North America and is also home to the most active pediatric lung transplant program in the country, rendering a diverse and large sample population. Therefore, we retrospectively reviewed the data of children with PAH who had genetic testing at Texas Children's Hospital (TCH) from October 2012 to August 2021. Diagnosis of PAH was made based on echocardiography and right heart catheterization when patients’ clinical status allowed.

### Genetic testing

The applied PAH gene panel included ACVRL1, BMPR2, CAV1, EIF2AK4, ENG, FLNA, GDF2, KCNA5, KCNK3, NOTCH1, NOTCH3, SMAD4, SMAD8, SOX17 and TOPBP1 genes. This panel was developed and administered by the John Welsh Cardiovascular Diagnostic Laboratory. Specifically, genomic DNA isolated from patient's blood sample was analyzed by sequencing for exons, splice junctions, and flanking regions of all genes tested in this panel. Sequencing analysis was performed by oligonucleotide-based in-solution hybridization target capture (SeqCap EZ, NimbleGen or KAPA HyperChoice, Roche) followed by next generation sequencing (MiSeq, Illumina). Sanger sequencing (3730XL DNA Analyzer, ABI) was used to fill in for gaps/bases that were not sufficiently covered. All clinically significant and novel variants were confirmed by independent Sanger sequencing. DNA sequence was assembled to and analyzed in comparison with the genomic reference sequences (GRCh37/hg19 or GRCh38/hg38) published in the NCBI database to generate variant calls. Variants were annotated through Esembl VEP program, curated *via* AlaMut and open source databases including gnomAD, ClinVar, VarSome and PubMed, and classified according to the ACMG guideline (12). Genetic variations were classified as pathogenic, variant with unknown significance (VUS) and polymorphisms (likely benign and benign). Further/correlating genetic testing was offered to parents as indicated.

### Clinical information

Clinical data correlates were obtained by reviewing medical records. Demographic data of the study patients, including age, gender, race, ethnicity, comorbidity and family history of PAH was collected. Clinical information including echocardiography findings, cardiac catheterization results, treatment combinations and outcome were also collected and analyzed. Presence of right ventricular failure was defined qualitatively based on the echo reports. Medical therapy was categorized as monotherapy, dual therapy and triple therapy.

### Statistical analysis

Data were analyzed using STATA 13.1 (StataCorp, College Station, TX). Summary statistics were described with mean and standard deviation for parametric variables and median with interquartile range (IQR) for nonparametric variables. Comparison of continuous variables in subgroups was performed using independent t-test or Wilcoxon rank-sum test, one-way analysis of variance, or Kruskal-Wallis depending on normality of distribution. Comparisons of categorical variables between subgroups were analyzed using the Chi-square test. The threshold for statistical significance was *p*-value <0.05.

## Results

There were 66 children with diagnosis of PAH who had genetic testing through John Welsh Laboratory during the study period (Table 1). Median (IQR) age of study patients was 4.4 (0.94, 11.4) years old. There was female predominance (*n* = 38, 58%). The majority of patients were Caucasian (*n* = 47, 71%) and 19 (40.4%) identified as Hispanic or Latino. The greater part of the study population (*n* = 40, 61%) had comorbidities either in the cardiac or respiratory system. Approximately 10% of children who underwent genetic testing had known family history of PAH.

**Table 1:** Demographics of patients by genetic testing results.

Genetic testing	Total (*n* = 66)	Pathogenic (*n* = 9)	Variant of unknown significance (*n* = 16)	Polymorphisms (likely benign and benign) (*n* = 41)
Median age at presentation (years), range	4.4 (0.94, 11.4)	7.6 (4.2, 14.4)	5.6 (1.2, 11.4)	3.0 (0.78, 8.9)
Male, *n* (%)	28 (42)	3 (33)	6 (38)	19 (46)
Race, *n* (%)				
Caucasian	47 (71)	8 (89)	10 (63)	29 (71)
African American	10 (15)	1 (11)	5 (31)	4 (10)
Asian	3 (5)	0 (0)	0 (0)	3 (7)
Other	6 (9)	0 (0)	1 (6)	5 (12)
Hispanic/Latino, *n* (%)	20 (30)	3 (33)	7 (44)	10 (24)
Family history of PAH, *n* (%)	7 (17)	2 (22)	2 (13)	3 (7)
WSPH group, *n* (%)				
Group 1	55 (83)	9 (100)	15 (94)	31 (73)
Group 2	3 (5)	0 (0)	1 (2)	2 (5)
Group 3	14 (21)	0 (0)	3 (5)	11 (27)
Group 4	0 (0)	0 (0)	0 (0)	0 (0)
Group 5	2 (3)	0 (0)	0 (0)	2 (5)
Comorbidities, *n* (%)	40 (61)	4 (44)	8 (50)	28 (68)
Cardiac disease	24 (36)	2 (22)	4 (25)	18 (434)
Pulmonary disease	25 (38)	2 (22)	4 (25)	19 (46)
Hematologic disease	2 (3)	0 (0)	0 (0)	2 (5)
Systemic disease	4 (6)	0 (0)	3 (19)	1 (2)
Metabolic disease	1 (2)	0 (0)	0 (0)	1 (2)

PAH, pulmonary arterial hypertension; WSPH, world symposium of pulmonary hypertension.

Nine children (14%) were found to have pathogenic mutations, 16 (24%) with variant of unknown significance and 41 (62%) with –polymorphisms (likely benign or benign) noted. Children with pathogenic mutations older than those without pathogenic mutations. BMPR2 mutation was the most common pathogenic mutation, seen in 6 children (Table 2). Other recorded mutations included EIF2AK4 pathogenic mutation, GDF2 pathogenic mutation in one child each respectively. And there was a child who was found to have TBX4 pathogenic mutation later on through a genetic testing out of our lab. The majority of the variants of unknown significance were located in the gene NOTCH1 (38%), followed by NOTCH3 (31%) and KCNA5 (31%).

**Table 2:** Identified mutations.

Mutation type, *n* (%)	Pathogenic (*n* = 9)	Variant of unknown significance (*n* = 16)
BMPR2	6 (67) c.1644delC (*p*.Ser549Profs*15) c.186dup (*p*.Gly63Argfs*2) c.2530C > T (*p*.Gln844*) c.189_190insT (*p*.Ser64*) c.1169delG (*p*.Gly390Glufs*3) c.978delA (*p*.Lys326Asnfs*9)	1 (6)
EIF2AK4	1 (11) c.301G > T (*p*.Glu101*) and c.1243delT (*p*.Tyr415Ifs*12)	1 (6)
ACVRL1	0 (0)	0 (0)
ENG	0 (0)	2 (13)
GDF2	1 (11) c.76C > T (*p*.Gln26*)	2 (13)
NOTCH1	0 (0)	6 (38)
NOTCH3	0 (0)	5 (31)
KCNA5	0 (0)	5 (3)
KCNK3	0 (0)	1 (6)
SMAD4	0 (0)	1 (6)
TBX4	1 (11.1) c.1164dup (*p*.Arg389Glnfs*30)	0 (0)
SOX17	0 (0)	1 (6.3)
TOPBP1	0 (0)	1 (6.3)

BMPR2, bone morphogenetic protein receptor 2; EIF2AK4, eukaryotic translation initiation factor 2-alpha kinase 4; ACVRL1, activin receptor like kinase 1; ENG, endoglin; GDF2, growth differentiation factor 2; NOTCH, neurogenic locus notch homolog protein; KCNA and KCNK, potassium channel subfamily A and K; T-box 4, T-box 4; SMAD4, suppressor of mothers against decapentaplegic 4; SOX17, SRY-related high-mobility group box; TOPBP1, DNA topoisomerase II binding protein 1.

Eighty-three percent of the study population was classified as World Symposium of Pulmonary Hypertension (WSPH) Group 1, WSPH Group 3 was 21% of patients. Children with features of Group 1 and Group 3 disease were classified in the WSPH group that corresponded to the driver of disease pathogenesis. There were 10 (15%) children who were classified as multifactorial disease (i.e., fit more than 1 WSPH classification), however none of them had pathologic mutations. All children with pathogenic mutations were classified as WHO group 1 (Group 1.2 for HPAH, 1.6 for PVOD). All children with pathogenic mutations had severe PAH requiring triple therapy (Table 3).

**Table 3:** Hemodynamic data and clinical outcome of patients by genetic testing results.

Genetic testing	Total (*n* = 66)	Pathogenic (*n* = 9)	Variant of unknown significance (*n* = 16)	Polymorphisms (likely benign and benign) (*n* = 41)	*p*-value*
Echocardiogram, *n* (%)	65/66 (99)	9/9 (100)	16/16 (100)	40/41 (98)	
Tricuspid regurgitant jet (m/sec)	4.6 (4.1, 5.1)	5.1 (4.5, 5.6)	4.6 (3.9, 5.2)	4.6 (3.9, 5.0)	0.22
Presence of right ventricular failure, *n* (%)	43/62 (66)	7/9 (78)	11/16 (69)	25/40 (63)	0.66
Cardiac catheterization, *n* (%)	46/66 (70)	8/9 (89)	13/16 (81)	25/41 (61)	
Pulmonary vascular resistance (Wood units)	4.6 (4.1, 5.1)	17.4 (11.1, 26.2)	4.6 (3.9, 5.2)	4.6 (3.9, 5.0)	0.17
Vasodilation study, *n* (%)	38/46 (81)	7/8 (88)	10/13 (77)	21/41 (51)	
Vasoreactivity, *n* (%)	14/38 (37)	3/7 (43)	5/10 (50)	6/21 (29)	0.48
Transplant, *n* (%)	10 (15)	3 (33)	3 (19)	4 (10)	0.15
Age at transplant (years), median (range)	13.6 (11.9, 15.4)	15.4 (11.8, 15.5)	13.3 (0.97, 13.9)	13.3 (12.3, 14.6)	0.60
Mortality, *n* (%)	15 (23)	1 (11)	5 (31)	9 (22)	0.53
Age at mortality (years), median (range)	11.5 (1.15, 17.0)	14.4 (14.4, 14.4)	11.5 (6.3, 17.0)	4.4 (0.90, 15.8)	0.64
Medical therapy^a^, *n* (%)					0.76
None	2 (3)	0 (0)	0 (0)	2 (5)	
Monotherapy	4 (6)	0 (0)	1 (6)	3 (7)	
Dual therapy	7 (11)	0 (0)	2 (13)	5 (12)	
Triple therapy	28 (42)	5 (56)	5 (3)	18 (44)	

^a^ Medical therapy excludes children who deceased or received lung transplantation.

* *p*-values are for 3 group comparison.

On echocardiogram, tricuspid regurgitant (TR) doppler signal was measured in 43 children. Median TR jet was elevated to 5.1 (4.5, 5.6) vs. 4.6 (3.9, 5.2) vs. 4.6 (3.9, 5.0) among children with pathogenic, VUS and polymorphism mutations respectively. The rate of RV failure was the highest in pathogenic mutation group [78% vs. 69% vs. 63%]. Cardiac catheterization revealed the highest pulmonary vascular resistance (PVR) with pathogenic mutations [17.4 (11.1, 26.2) vs. 4.6 (3.9, 5.2) vs. 4.6 (3.9, 5.0)]. About half of the children with pathogenic and VUS mutations had positive vasoreactivity during cardiac catheterization (43%, 50%). Vasoreactivity was least observed among those with polymorphism mutations (29%).

Outcomes for the cohort with identified pathogenic mutation trended to higher rates of transplantation, 33% (*n* = 3) compared to those without pathogenic mutations (*n* = 7, 12%). The age of lung transplantation was similar at 13–15 years old among all genetic groups. The median (IQR) time to the lung transplant was 9.8 (1.2, 72.0) months ranging from 0.78 months to 12.5 years.

Mortality was higher in the non-pathogenic group (VUS and polymorphism composite) compared to the group of patients with pathogenic mutations (*n* = 14, 53% vs. *n* = 1, 11%, *p* = 0.53). The one death in the pathogenic mutation group was a 14 year-old child who died during diagnostic admission; later discovered to have BMPR2 mutation.

Genetic testing was offered to four families with known pathogenic mutation and two families with detected variant of unknown significance. Inherited pathogenic mutations were identified in two families of patients with pathogenic mutation. The first family had a mutation in EIF2AK4. The parents had c.1243delT (*p*.Tyr415Ifs*12) and c.301G > T (*p*.Glu101*) and the child inherited both of these mutations. The second family had a mutation in GDF2: both of the parents had c.76C > T (*p*.Gln26*), which the child inherited. In families with children who resulted with variant of unknown significance, no no pathogenic mutations were identified.

## Discussion

We describe the genetic burden of disease within a large pediatric referral center for PH. In our cohort, we identified PAH-associated pathogenic genetic mutations in 13.6% of the patients who otherwise had no obvious etiology for their disease. This is lower than previous studies reporting 20%–50% prevalence of genetic mutations in pediatric onset PAH (3, 13, 14). One of the possible explanations is limitations on our genetic panel not including newer PAH genes such as BMP10, aquaporin 1 (AQP1), ATPase 13A3 (ATP13A3) or kinase insert domain receptor (KDR). BMPR2 mutation was the most common mutation seen among 66.7% of mutations in our population, consistent with the literature. BMPR2 mutation has been reported as the most common mutation in both pediatrics and adults, comprising 6.5%–12.5% of all unexplained pediatric PAH and 36%–65% of pediatric PAH with pathogenic genetic variants (3, 5, 13, 15).

In our patient population, higher TR jet and more RV failure was noted on initial echocardiograms among children with pathogenic mutation, suggesting more clinical burden of disease (16). This trend was confirmed by cardiac catheterization, showing higher PVR among children with pathogenic mutations compared to those without them. PVR among children with pathogenic mutation in our population was similar to the previously reported values of 19.9 WU among children with idiopathic and heritable PAH by Zhang et al. (14). All children with pathologic mutation had severe PAH at presentation and all were started on upfront combination therapy. Outcomes for HPAH in our population were commensurate with reports of severe disease in the literature, including high rate of transplantation. It is interesting to note that the mortality in the HPAH cohort was overall less than the non-HPAH cohort. This may reflect the benefit of upfront aggressive medical treatment that was offered to the group with pathogenic mutation and the expectant referral provided for transplantation in the face of poor prognosis. Although the majority of our population did not have an identified pathogenic mutation on targeted screening, this non-genetic disease group did demonstrate progressive and aggressive disease, consistent with idiopathic PAH, WSPH Group 1 (86%). This percentage is higher than literature reports of 58% idiopathic in the pediatric population (3). This discrepancy may reflect a limitation in our institutional genetic panel or reflect the diversity of our patient population. Advancement in genetic studies including identification of new genetic mutations is required for these patient population.

A previous population-based study among insured United States (US) patients reported that 36% of pediatric PAH patients had a history of prematurity, 75% with congenital heart defect and 13% with trisomy 21 (17). In this selected population that underwent genetic testing, only 8 (12%) children had documented history of prematurity, 18 (27%) had cardiac defect and none of the referred patients had other known PAH associated genetic syndromes such as trisomy 21. This difference is likely reflective of some sample bias as genetic studies were only offered for unexplained or disproportionate PH symptoms.

In addition to the limitations already stated, there was insufficient data on familial testing to report on the benefit of that modality in this study. Although pathogenic mutations were discovered and group classification was changed to WSPH Group 1.2, further familial studies were necessary to confirm hereditary nature of the discovered mutations. Secondly, the overall number of children with pathogenic mutation was small. This is likely a limitation of targeted or known familial mutation testing. Application of broader panels will likely find additional genetic abnormalities. Finally, due to the retrospective nature of the study, our data was limited for data prior to presentation at our center and to testing available during a specific era. Genetic panels are evolving quickly and negative testing in 2012 may not be negative on repeat, expanded testing. The panel that was used for genetic testing in this population was updated over time by adding new genes. Although BMPR2 has been on the panel since testing was started, and this diagnostic yield was not significantly affected over study period, it is possible that mutations in other genes of interest were missed in early testing.

We recognize that the findings in this study may be unique and not be generalizable for the greater pediatric PAH population. However, we hope that this descriptive study informs on the genetic burden of disease in pediatric PH patients and the consideration for genetic screening to affect morbidity and mortality. Certainly, larger population studies and registries for pediatric PAH are warranted to better characterize the greater burden of HPAH and phenotype-genotype correlations.

## Conclusion

Genetic mutations are not uncommon among children with PAH. Children affected with pathogenic mutations presented with more severe PAH, higher pulmonary vascular resistance and higher rate of RV failure requiring upfront triple therapy. These children had higher rates of transplantation but improved survival, perhaps because of more aggressive treatment in the setting of known HPAH. Genetic testing should be routinely considered due to consequences for treatment and prognostic implications. Further studies in larger population and registries are warranted to better characterize pediatric HPAH.

## Data Availability

The datasets presented in this study can be found in online repositories. The names of the repository/repositories and accession number(s) can be found below: https://www.ncbi.nlm.nih.gov/, SCV002575026 - SCV002575059.
